# MRI of the chest: review of imaging strategies

**DOI:** 10.1590/0100-3984.2015.48.6e1

**Published:** 2015

**Authors:** Carolina A. Souza

**Affiliations:** 1 MD, PhD, Thoracic Radiologist, The Ottawa Hospital, Associate Professor, University of Ottawa Clinical Investigator, The Ottawa Hospital Research Institute, Ottawa, Canada. E-mail: carolina_althoff@yahoo.ca.

Magnetic resonance imaging (MRI) is an imaging modality widely used in clinical practice. The use of MRI for imaging of the thorax, however, has been historically considered of limited value, despite the effort of physicists and radiologists to obtain positive and reproducible results in several studies. MRI plays a role in the assessment of cardiovascular disease, mediastinal lesions and abnormalities of the brachial plexus and chest wall. However, clinical indications are restricted to specific conditions, generally as a problem-solving technique.

Continuous motion from cardiac and vascular pulsation and respiratory motion are one of the major challenges in MRI of the chest as they severely affect imaging quality. A significant limitation of thoracic MRI is imaging of the lung due to intrinsic characteristics of the pulmonary tissue and the presence of physiologic motion. The low proton density of the lung parenchyma generates low signal intensity and low signal-to-noise ratio when compared to other parts of the body. Furthermore, susceptibility artifacts at tissue-air and liquid-air interfaces of the alveoli greatly affect signal intensity^(1,2)^. In more recent years, however, MRI has evolved from a research tool to a useful modality in the assessment of thoracic disease. Technical advances such as very short echo times and ultrafast turbo-spin-echo acquisitions, that allow breath-hold imaging with full anatomic coverage and help to overcome cardiac pulsation^(3,4)^, have improved the capability of thoracic MRI. The use of contrast agents for perfusion MRI and gas imaging for assessment of pulmonary ventilation have further increased the applications of MRI in the investigation of lung diseases^(5-8)^.

In the article "Chest magnetic resonance imaging: a protocol suggestion", published in the current issue of **Radiologia Brasileira**, Hochhegger et al.^(9)^ provide a concise yet comprehensive review of MRI of the chest. The authors convey an instructive discussion of technical aspects and challenges of thoracic MRI, including limitations of 3T MRI in the chest, and suggest strategies to overcome some of these obstacles.

In the first part of the manuscript, the main clinical indications of MRI of the chest is presented, including recent data regarding the role of MRI in the distinction between malignant and benign pulmonary nodules^(10,11)^ as well as the advantages of MRI in the staging of lung cancer when compared to CT and 18-FDG-PET/CT. Various studies have demonstrated the value of MRI in the detection and assessment of degree of tumor invasion in the mediastinum, pleura and chest wall, thus contributing to the T descriptor of the TNM staging system^(12-14)^. The present article discuss the role of whole-body diffusion-weighted MRI in the detection of distant metastases (M staging) and remark the potential role of diffusion MRI in the characterization of irradiated lung tissue^(15)^. The authors also describe the advantages of MRI in the morphological and functional assessment of patients with pulmonary hypertension, including estimation of cardiac function as well as surgical planning^(16,17)^. Importantly, the use of MRI in the diagnosis of pulmonary embolism and in the follow up of cystic fibrosis and pneumonia are discussed^(18-20)^, highlighting the importance of MRI in populations at increased risk of ionizing radiation complications such as young patients and in pregnancy. Recent studies assessing the use of thoracic MRI in cystic fibrosis in particular have shown promising results, including depiction of morphological changes as well as evaluation of pulmonary function and respiratory mechanics^(18,21,22)^. The clinical impact of an accurate ionizing radiation-free modality for a young population requiring frequent imaging assessment cannot be underestimated.

The authors finalize the article providing a basic MRI protocol applicable to most common thoracic diseases while also suggesting additional sequences to be used in specific thoracic abnormalities. The proposed protocols are suitable to most state-of-art MRI scanners and can be easily implemented.

I commend the authors for they effort to present MRI as a valuable modality in the assessment of pulmonary diseases, increasing awareness of the peculiarities and advantages of this modality to the radiology community as well as for providing objective tools that can move MRI from an underutilized technique to a modality with the potential to substantially contribute to thoracic radiology.
